# A visual positioning model for UAV’s patrolling video sequence images based on DOM rectification

**DOI:** 10.1038/s41598-023-49001-8

**Published:** 2023-12-07

**Authors:** Haojie Liu, Wei Fan, Di Wu

**Affiliations:** Yellow River Engineering Consulting Co., LTD., Zhengzhou, 450003 China

**Keywords:** Civil engineering, Information technology, Imaging and sensing

## Abstract

With technological development of multi sensors, UAV (unmanned aerial vehicle) can identify and locate key targets in essential monitoring areas or geological disaster-prone areas by taking video sequence images, and precise positioning of the video sequence images is constantly a matter of great concern. In recent years, precise positioning of aerial images has been widely studied. But it is still a challenge to simultaneously realize precise, robust and dynamic positioning of UAV’s patrolling video sequence images in real time. In order to solve this problem, a visual positioning model for patrolling video sequence images based on DOM rectification is proposed, including a robust block-matching algorithm and a precise polynomial-rectifying algorithm. First, the robust block-matching algorithm is used to obtain the best matching area for UAV’s video sequence image on DOM (Digital Orthophoto Map), a pre-acquired digital orthophoto map covering the whole UAV’s patrolling region. Second, the precise polynomial-rectifying algorithm is used to calculate accurate rectification parameters of mapping UAV’s video sequence image to the best matching area obtained above, and then real time positioning of UAV’s patrolling video sequence images can be realized. Finally, the above two algorithms are analyzed and verified by three practical experiments, and results indicate that even if spatial resolution, surface specific features, illumination condition and topographic relief are significantly different between DOM and UAV’s patrolling video sequence images, proposed algorithms can still steadily realize positioning of each UAV’s patrolling video sequence image with about 2.5 m level accuracy in 1 s. To some extent, this study has improved precise positioning effects of UAV’s patrolling video sequence images in real time, and the proposed mathematical model can be directly incorporated into UAV’s patrolling system without any hardware overhead.

## Introduction

Patrol inspection is an important work in many industries, and UAV (unmanned aerial vehicle) has gradually become a new tool for field patrol inspection due to its low cost and high efficiency. Shooting video to discover what has happened at sometime and somewhere in real-time is a common way of field patrol inspection by UAV, which is usually equipped with an image sensor and a POS (position and orientation system) unit. Accessing precise location of interest points in UAV’s real time patrolling video sequence images is of great value for discovery and elimination of hidden safety hazards. At present, there are four different kinds of methods that can be used for positioning of UAV’s patrolling video sequence images: photogrammetry method, binocular vision method, image feature matching method, and optical flow method.

Photogrammetry method includes forward intersection algorithm and block adjustment algorithm that can be both used for positioning of UAV’s patrolling video sequence images. Forward intersection algorithm^1–3^ can calculate out geodetic coordinates of homologous image points with assistance of POS data in real time. But the poor results cannot meet accuracy requirements of UAV’s patrolling video sequence images positioning. Block adjustment algorithm^4–6^ can precisely calculate out geodetic coordinates of homologous image points by using large overlapped sequence images under certain geometric conditions, but it is a post-processing algorithm which cannot meet the real-time requirements of UAV’s patrolling video sequence images positioning.

Binocular vision method uses binocular cameras with precise 3D coordinates and 3D orientations to shoot two images of the same scene simultaneously, and then geodetic coordinates of homologous image points can be calculated out according to vertical parallax of the two images. Binocular vision method has high accuracy and efficiency, and mainly focus on precise calibration of the fixed binocular cameras at present^7–13^, which cannot meet the dynamic requirement of UAV’s patrolling video sequence images positioning.

Feature matching method realizes image matching by verifying consistency of descriptors that are obtained from surrounding pixels of corresponding key-points in two images. The famous SIFT (scale invariant feature transform) algorithm realizes feature matching of scale-invariant, rotation-invariant and illumination-invariant by constructing Gaussian pyramid images and regional gradient distributions^14,15^, which is widely employed in image registration^16,17^ and image mosaic^18,19^. Ke proposed PCA-SIFT algorithm^20^ by using PCA (Principal Component Analysis) method to reduce dimension of regional gradient distributions, which improved efficiency of feature matching to a certain extent. Following the idea of scale-invariant in SIFT, Morel proposed a so-called ASIFT algorithm of affine-invariant by simulating image geometric distortions caused by variations of camera optical axis^21^. Bay constructed multi-scale spaces by using box filters and integral images, constructed key-points and corresponding descriptors by using non-maximum suppression method^22^ and Haar wavelet transform, and then proposed SURF (Speed Up Robust Features) algorithm^23,24^. SURF is one order magnitude faster than SIFT^25^. A more faster algorithm ORB (Oriented FAST and Rotated BRIEF)^25^, further proposed by Bblee, used oriented FAST (Features from Accelerated Segment Test) algorithm^26^ to detect key-points and used rotated BRIEF (Binary Robust Independent Elementary Features) algorithm^27^ to construct descriptors. Other feature matching methods also exert certain influence on image matching, including BRISK (Binary Robust Invariant Scalable Keypoints) algorithm^29^, KAZE algorithm^30^, hardware acceleration algorithm^31^, and etc. Literature^32,33^ compare and analyze accuracy, efficiency, advantages and disadvantages of existing feature matching methods in details, and we will not go into much here. Feature matching method can be used for precise real-time image matching, while matching results only have relative positioning information, which cannot meet the absolute positioning requirements of UAV’s patrolling video sequence images.

Optical flow method can obtain motion displacement of pixels between two adjacent sequence images through energy differential-difference equations which are constructed by certain assumptions and solved by certain optimization criteria. If all the image pixels are involved in this method, we call it dense optical flow, and if only part of the image pixels are involved in this method, we call it sparse optical flow. Two of the most classical optical flow algorithms are LK optical flow^34^ and HS optical flow^35^. LK optical flow is constructed on three basic assumptions, namely, brightness constancy (projection of the same point looks the same in every frame), small motion (points do not move very fast) and spatial coherence (points move like their neighbors)^34^. LK optical flow can calculate out motion displacement of pixels between two adjacent sequence images accurately, but performs poor stability sometimes. Based on above mentioned three basic assumptions, HS optical flow adds a regularization term in the self-constructed differential-difference equations. By minimizing the self-constructed differential-difference equations with regularization term, HS optical flow obtains the optimal motion displacement between two adjacent sequence images’ homologous points, which achieves a more stable performance. However, “brightness constancy” and “small motion” are two strong assumptions in LK optical flow and HS optical flow, which are difficult to be satisfied in practical applications. For this reason, lots of improved algorithms have been proposed. In Literature^36^, gradient conservation is used to replace the assumption of brightness constancy, which improves robustness of optical flow algorithm against illumination variation. Literature^37^ proposes multi-scale searching strategies, which has improved optical flow algorithm’s tracking efficiency of objects with large motion and shortened calculating time. A coarse-to-fine process has been mentioned in literature^38^, which further improves optical flow algorithm’s tracking ability of objects with large motion. Literature^39^ proposes an optical flow algorithm based on interpolation of correspondences, which has achieved good results in tracking objects with large displacement and significant occlusions. In literature^40,41^, polynomials fitted by intensity of regional pixels are used for tracking objects with large motion, illumination variation and noise interference, and good results have also been achieved. With the development of artificial intelligence, optical flow algorithm based on neural network^42^ has also emerged, but their robustness on unknown data sets remains to be verified. At present, optical flow algorithm has been widely used in medical image registration^44,45^, remote sensing image registration^46^, visual navigation^47^ and many other industries. Optical flow method can be used for precise matching of sequence images, while the relative positioning results cannot meet the absolute positioning requirements of UAV’s patrolling video sequence images.

To sum up, there is no method that can solve absolute positioning of UAV’s patrolling video sequence images accurately and robustly in real time. For this reason, a series of visual positioning algorithms for UAV’s patrolling video sequence images based on DOM rectification are proposed following the coarse-to-fine principle in this paper. All the proposed algorithms are analyzed and verified by three practical experiments, and results show that these algorithms are fast, effective and feasible.

## Methodology

### Technical flow

**Figure 1:**
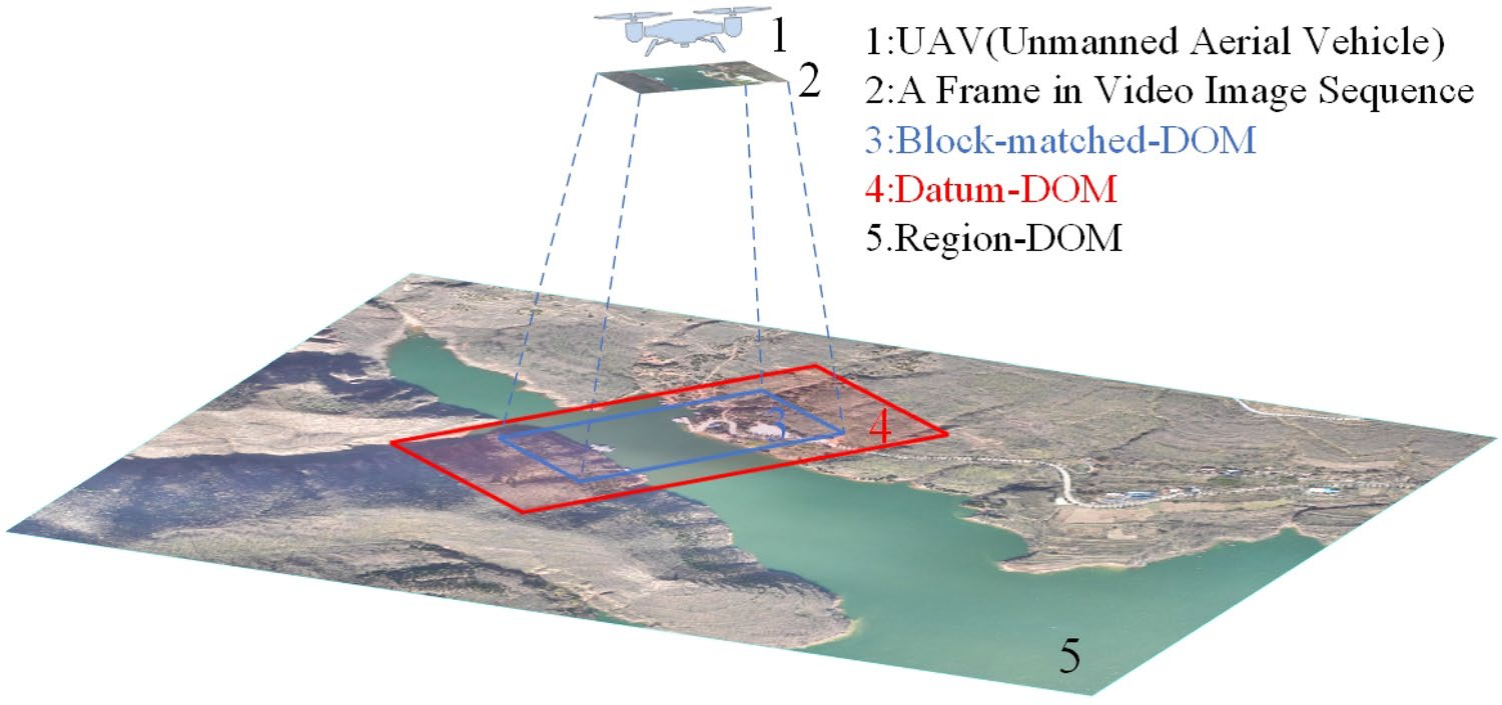
Key images involved in this study.

As shown in Fig. 1, number 1 is a UAV (unmanned aerial vehicle) in patrolling; Number 2 is a UAV’s video sequence image, which is taken by the patrolling UAV (number 1) and is needed to be positioned in real time; Number 3 is named as region-DOM, which is a digital orthophoto map of UAV’s patrolling region and is produced in advance; Number 4 is named as datum-DOM, which is a subarea of region-DOM (number 3); Number 5 is named as block-matched-DOM, which is further a subarea of datum-DOM (number 4) and is the best matching region for UAV’s video sequence image (number 2) on datum-DOM (number 4). It should be noted that, UAV’s patrolling video sequence image is abbreviated as video frame for convenience of subsequent work.

As shown in Fig. 1, the basic idea of this paper is to find out the best matching region (number 5) for video frame (number 2) on region-DOM (number 3) quickly and robustly, figure out the accurate rectification parameters for mapping video frame (number 2) to the best matching region (number 5), and finally realize real time positioning of video frame (number 2) by using accurate rectification parameters to obtain geodetic coordinates of each pixels in video frame (number 2). Following the basic idea and the coarse-to-fine principle, the technical flow of this study is described as follows.

First, extract datum-DOM (number 4) from region-DOM (number 3) according to the POS data of video frame (number 2), and replace region-DOM (number 3) by datum-DOM (number 4) as a new matching area for video frame (number 2), so as to reduce matching area of video frame (number 2) on region-DOM (number 3) and increase matching speed.

Second, extract block-matched-DOM (number 5) from datum-DOM (number 4) by using the proposed robust block-matching algorithm. It should be noted that, video frame (number 2) and block-matched-DOM (number 5) have the same size in pixels, but the matching accuracy between these two images is still poor due to numerous negative factors. Therefore, a further optimization step is needed.

Third, figure out accurate rectification parameters for mapping video frame (number 2) to block-matched-DOM (number 5) by using the proposed precise polynomial-rectifying algorithm.

Finally, obtain geodetic coordinates of each pixel in video frame (number 2) by using the accurate rectification parameters calculated above, so as to realize the real time positioning of video frame (number 2).

### Algorithm framework

**Figure 2:**
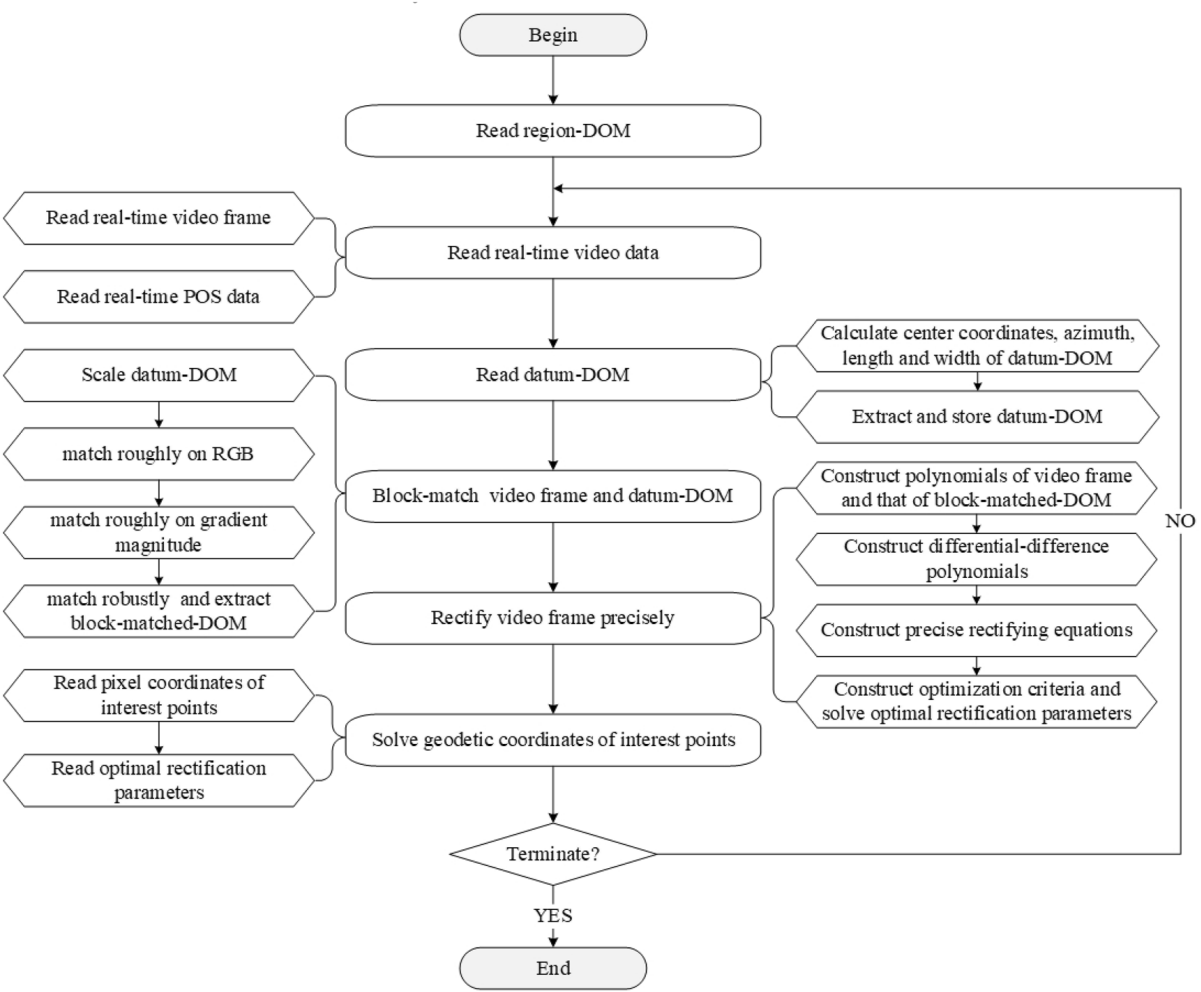
Algorithm framework.

The algorithm flow of this study is shown in Fig. 2. Advantages lie in the proposed robust image-block-matching algorithm and precise polynomial-rectifying algorithm, which can solve geodetic coordinates of all pixels in a UAV’s real-time video frame with about 2.5 m level accuracy in 1 s.

## The visual positioning model

### Extraction of datum-DOM

Following the basic idea of this paper, datum-DOM should be extracted from region-DOM at the beginning, so as to reduce matching area of video frame on region-DOM and increase matching speed. As shown in Fig. 1, Central point’s coordinates of datum-DOM is determined by geodetic coordinates of UAV’s POS data; Azimuth of datum-DOM is determined by yaw angle of UAV’s POS data; Length and width of datum-DOM in pixels is determined by equations as:

1 $$\left\{\begin{array}{c}{L}_{pixels}=n\frac{{L}_{dist}}{{gsd}_{D}}\\ {W}_{pixels}=n\frac{{W}_{dist}}{{gsd}_{D}}\end{array}\right.$$

where, ${L}_{pixels}$ and ${W}_{pixels}$ are length and width of datum-DOM in pixels respectively; ${L}_{dist}={H}_{fly}{\times L}_{CMOS}/f$; ${W}_{dist}={H}_{fly}{\times W}_{CMOS}/f$; ${L}_{CMOS}$ and ${W}_{CMOS}$ are physical length and width of UAV’s CMOS (Complementary Metal Oxide Semiconductor) sensor respectively; $f$ is focal length of UAV’s camera; ${gsd}_{D}$ is spatial resolution of datum-DOM; $n$ is scaling coefficient, ranging from 1.5 to 2.

Finally, datum-DOM can be extracted from region-DOM according to the already known parameters $\left({L}_{POS},{B}_{POS},{Yaw}_{pos},{L}_{pixels},{W}_{pixels}\right)$. Where, $\left({L}_{POS},{B}_{POS}\right)$ are central point’s coordinates of datum-DOM; ${Yaw}_{pos}$ is yaw angle of UAV’s POS data; ${L}_{pixels}$ and ${W}_{pixels}$ are obtained from Eq. (1).

### Construction of robust block-matching algorithm

Follow the basic idea of this paper, the best matching area for video frame on datum-DOM should be extracted. However, existing image feature matching methods are all difficult to match video frame and datum-DOM automatically, since illumination conditions, surface specific features, projection modes and spatial resolution of these two kinds images are greatly different. Therefore, a robust block-matching algorithm is constructed for the purpose of finding out the best matching area for video frame on datum-DOM.

#### Scaling datum-DOM

It is necessary to unify spatial resolutions of datum-DOM and video frame, so as to facilitate the subsequent matching work. To ensure the spatial resolution of datum-DOM is the same as video frame, datum-DOM is scaled as:

2 $${img}_{{D}_{S}}={img}_{D}\cdot {Scale}_{D}$$

where ${img}_{{D}_{S}}$ represent the size of scaled datum-DOM; ${img}_{D}$ represent the size of original datum-DOM; ${Scale}_{D}={gsd}_{D}/{gsd}_{F}$; ${gsd}_{D}$ is spatial resolution of the original datum-DOM; ${gsd}_{F}$ is spatial resolution of video frame;

#### Block-matching roughly based on RGB color

**Figure 3:**
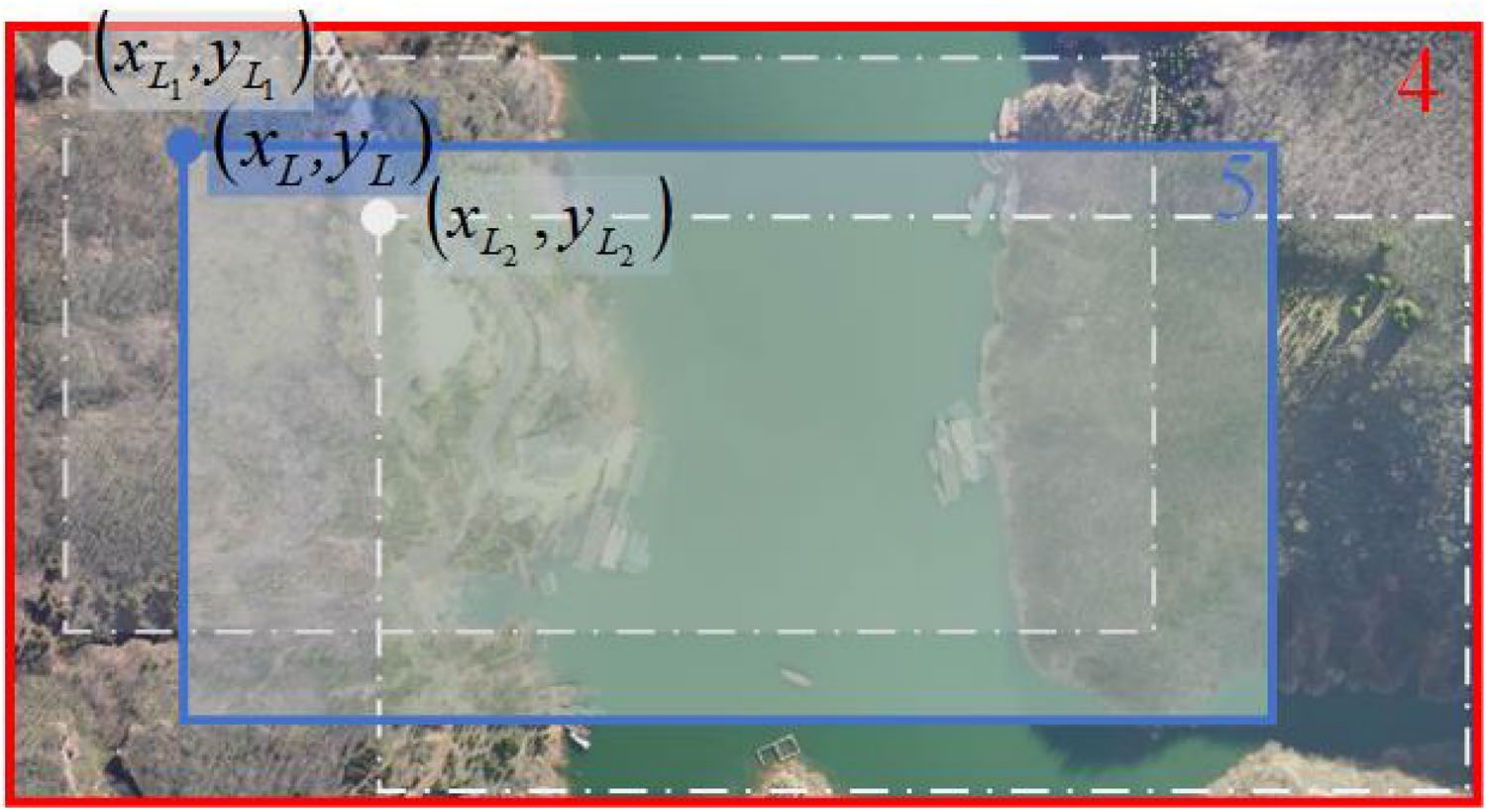
The robust block-matching of datum-DOM and video frame.

At this step, the best matching area for video frame on datum-DOM can be found out based on the similarity of these two images in RGB color space. As shown in Fig. 3, $({x}_{L1},{y}_{L1})$ are pixel coordinates of the top left corner of the best matching area for video frame on datum-DOM in RGB color space, and $({x}_{L1},{y}_{L1})$ can be obtained as:

3 $$\left({x}_{L1},{y}_{L1}\right)=\underset{\mathit{arg}({\Delta x}_{1},{\Delta y}_{1})}{\mathit{max}}\frac{{\sum }_{x,y}{F}_{R}{D}_{R}+{\sum }_{x,y}{F}_{G}{D}_{G}+{\sum }_{x,y}{F}_{B}{D}_{B}}{\sqrt{{\sum }_{x,y}{F}_{R}^{2}{\sum }_{x,y}{D}_{R}^{2}}+\sqrt{{\sum }_{x,y}{F}_{G}^{2}{\sum }_{x,y}{D}_{G}^{2}}+\sqrt{{\sum }_{x,y}{F}_{B}^{2}{\sum }_{x,y}{D}_{B}^{2}}}$$

where, $$\left\{\begin{array}{l}{F}_{R}={F}_{{R}_{src}}(x,y)-\sum_{x,y}{F}_{{R}_{src}}(x,y)/{N}_{F}\\ {D}_{R}={D}_{{R}_{src}}({\Delta x}_{1}+x,{\Delta y}_{1}+y)-\sum_{x,y}{D}_{{R}_{src}}({\Delta x}_{1}+x,{\Delta y}_{1}+y)/{N}_{F}\\ \begin{array}{l}{F}_{G}={F}_{{G}_{src}}(x,y)-\sum_{x,y}{F}_{{G}_{src}}(x,y)/{N}_{F}\\ \begin{array}{l}{D}_{G}={D}_{{G}_{src}}({\Delta x}_{1}+x,{\Delta y}_{1}+y)-\sum_{x,y}{D}_{{G}_{src}}({\Delta x}_{1}+x,{\Delta y}_{1}+y)/{N}_{F}\\ {F}_{B}={F}_{{B}_{src}}(x,y)-\sum_{x,y}{F}_{{B}_{src}}(x,y)/{N}_{F}\\ {D}_{B}={D}_{{B}_{src}}({\Delta x}_{1}+x,{\Delta y}_{1}+y)-\sum_{x,y}{D}_{{B}_{src}}({\Delta x}_{1}+x,{\Delta y}_{1}+y)/{N}_{F}\end{array}\end{array}\end{array}\right.$$;${F}_{{R}_{src}}(x,y)$, ${F}_{{G}_{src}}(x,y)$ and ${F}_{{B}_{src}}(x,y)$ are intensity of $R$, $G$ and $B$ channel of video frame respectively; ${D}_{{R}_{src}}({\Delta x}_{1}+x,{\Delta y}_{1}+y)$, ${D}_{{G}_{src}}({\Delta x}_{1}+x,{\Delta y}_{1}+y)$ and ${D}_{{B}_{src}}({\Delta x}_{1}+x,{\Delta y}_{1}+y)$ are intensity of $R$, $G$ and $B$ channel of datum-DOM respectively; $(x,y)$ are pixel coordinates in video frame, $x=(\mathrm{1,2},\cdots ,{N}_{LF})$, $y=(\mathrm{1,2},\cdots ,{N}_{WF})$; $({\Delta x}_{1},{\Delta y}_{1})$ are pixel coordinates of video frame’s top left corner in datum-DOM, ${\Delta x}_{1}=(\mathrm{1,2},\cdots ,{N}_{LD}-{N}_{LF})$, ${\Delta y}_{1}=(\mathrm{1,2},\cdots ,{N}_{WD}-{N}_{WF})$ ; ${N}_{LF}$ and ${N}_{WF}$ are length and width of video frame in pixels respectively; ${N}_{LD}$ and ${N}_{WD}$ are length and width of datum-DOM in pixels respectively; ${N}_{F}$ is the total pixel numbers of video frame, ${N}_{F}={N}_{LF}{N}_{WF}$.

#### Block-matching roughly based on gradient magnitude

At this step, the best matching area for video frame on datum-DOM can be found out based on the similarity of these two images in gradient magnitude space. As shown in Fig. 3, $({x}_{L2},{y}_{L2})$ are pixel coordinates of the top left corner of the best matching area for video frame on datum-DOM in gradient magnitude space, and $({x}_{L2},{y}_{L2})$ can be obtained as:

4 $$({x}_{L2},{y}_{L2}) =\underset{\mathit{arg}({\Delta x}_{2},{\Delta y}_{2})}{\mathit{max}}\frac{{\sum }_{x,y}{\left[{I}_{F}\left(x,y\right){I}_{D}({x}_{L2}+x{,y}_{L2+y})\right]}^{2}}{\sqrt{{\sum }_{x,y}{I}_{F}^{2}\left(x,y\right){\sum }_{x,y}{I}_{D}^{2}({x}_{L2}+x{,y}_{L2+y})}}$$

where,

$$\left\{\begin{array}{l}{I}_{F}(x,y)=\sqrt{{F}_{x}^{2}(x,y)+{F}_{y}^{2}(x,y)} \\ {I}_{D}({\Delta x}_{2}+x,{\Delta y}_{2}+y)=\sqrt{{D}_{x}^{2}({\Delta x}_{2}+x,{\Delta y}_{2}+y)+{D}_{y}^{2}({\Delta x}_{2}+x,{\Delta y}_{2}+y)}\end{array}\right.$$; ${F}_{x}(x,y)$ and ${F}_{y}(x,y)$ are first partial derivative of video frame in $x$ and $y$ direction respectively; ${D}_{x}({\Delta x}_{2}+x,{\Delta y}_{2}+y)$ and ${D}_{y}({\Delta x}_{2}+x,{\Delta y}_{2}+y)$ are first partial derivative of datum-DOM in $x$ and $y$ direction respectively; $(x,y)$ are pixel coordinates in video frame, $x=(\mathrm{1,2},\cdots ,{N}_{LF})$, $y=(\mathrm{1,2},\cdots ,{N}_{WF})$; $({\Delta x}_{2},{\Delta y}_{2})$ are pixel coordinates of video frame’s top left corner in datum-DOM, ${\Delta x}_{2}=(\mathrm{1,2},\cdots ,{N}_{LD}-{N}_{LF})$, ${\Delta y}_{2}=(\mathrm{1,2},\cdots ,{N}_{WD}-{N}_{WF})$; ${N}_{LF}$ and ${N}_{WF}$ are length and width of video frame in pixels respectively; ${N}_{LD}$ and ${N}_{WD}$ are length and width of datum-DOM in pixels respectively;

#### Block-matching robustly

In practice, it has been found that the above proposed RGB based block-matching method exhibits better performance in video frame with large color difference and complicate texture, while the above proposed gradient magnitude based block-matching method exhibits better performance in video frame with small color difference and simple texture.Therefore, it is necessary to further construct a robustly block-matching method by considering both color difference and texture complexity of video frame.

In the robustly block-matching method, symbol $TH$ is proposed to comprehensive represent color difference amplitude and texture complexity of video frame, and a threshold number 20 is selected to judge $TH$. If $TH\le 20$, the video frame is considered to have large color difference and complicate texture, and the matching result in section "Block-matching Roughly Based on RGB color" should have a lager weight. On the contrary, if $\mathrm{TH}>20$, the video frame is considered to have small color difference and simple texture, and the matching result in section "Block-matching Roughly Based on Gradient Magnitude" should have a larger weight. $TH$ is calculated in Eq. (5), and the threshold number 20 is selected by numerous practical experiments.

As shown in Fig. 3, $({x}_{L},{y}_{L})$ are coordinates of the top left corner of the best matching area obtained by the proposed robustly block-matching method, and $({x}_{L},{y}_{L})$ can be calculated as:

5 $$\left({x}_{L},{y}_{L}\right)=\left\{\begin{array}{c}\frac{\left[\left(\genfrac{}{}{0pt}{}{{x}_{{L}_{1}}}{{y}_{{L}_{1}}}\right)+{\omega }_{L}\left(\genfrac{}{}{0pt}{}{{x}_{{L}_{2}}}{{y}_{{L}_{2}}}\right)\right]}{1+{\omega }_{L}} \,\,\,\,\,\,\,\,\,TH\le 20\\ \frac{\left[{\omega }_{L}\left(\genfrac{}{}{0pt}{}{{x}_{{L}_{1}}}{{y}_{{L}_{1}}}\right)+\left(\genfrac{}{}{0pt}{}{{x}_{{L}_{2}}}{{y}_{{L}_{2}}}\right)\right]}{1+{\omega }_{L}}  \,\,\,\,\,\,\,\,\,TH>20\end{array}\right.$$

$$TH=\sum_{x,y}\left|{I}_{F}(x,y)-{I}_{D}({x}_{{L}_{2}}+x,{y}_{{L}_{2}}+y)\right|/{N}_{F}$$

where, ${\omega }_{L}$ is a weight, $${\omega }_{L}=\left\{\begin{array}{l}1,\quad r\le 1/2\\ 1/2r,\quad 1/2<r\le 1\\ 0,\quad 1<r\end{array}\right.$$, $r$ represents a distance between $\left({x}_{{L}_{1}},{y}_{{L}_{1}}\right)$ and $\left({x}_{{L}_{2}},{y}_{{L}_{2}}\right)$, $r=\frac{\sqrt{{\left({x}_{{L}_{1}}-{x}_{{L}_{2}}\right)}^{2}+{\left({y}_{{L}_{1}}-{y}_{{L}_{2}}\right)}^{2}}}{\sqrt{{\left({N}_{LF}/10\right)}^{2}+{\left({N}_{WF}/10\right)}^{2}}}$, equations of $r$, ${\omega }_{L}$ and $TH$ are all constructed by numerous practical experiments; $\left({x}_{{L}_{1}},{y}_{{L}_{1}}\right)$ and $\left({x}_{{L}_{2}},{y}_{{L}_{2}}\right)$ are obtained by Eqs. (3) and (4) respectively; ${N}_{LF}$ and ${N}_{WF}$ are length and width of video frame in pixels respectively; ${N}_{F}$ is the total pixel numbers of video frame; TH represents color difference and texture complexity of video frame; the threshold number 20 is selected by numerous practical experiments; Meaning of the rest parameters can refer to Eqs. (3) and (4).

#### Extracting block-matched-DOM

According to parameters ${(x}_{L},{y}_{L},{N}_{LF},{N}_{WF})$ calculated in Eq. (5), Block-matched-DOM can be extracted from datum-DOM. As shown in Fig. 3, block-matched-DOM is the area in blue box marked by number 5, and is the best matching area for video frame on datum-DOM ultimately found.

It should be noted that, video frame and its corresponding block-matched-DOM have the same size in pixels, and geodetic coordinates of each pixel on video frame can be obtained directly from the geodetic coordinates of pixels at the same position on block-matched-DOM. That is to say, positioning of UAV’s patrolling video frame can be realized by directly assigning geodetic coordinates of each pixel in block-matched-DOM to pixels at the same position in UAV’s patrolling video frame.

### Construction of precise polynomial-rectifying algorithm

Unfortunately, there is a high probability that pixels in video frame are not homologous with pixels in block-matched-DOM at the same position, due to numerous negative factors, such as illumination variation, inconsistent spatial resolution, diverse surface specific features, topographic relief, camera distortion, different projection modes and etc. That is to say, the positioning accuracy of video frame is still poor, if we assign geodetic coordinates of each pixel in block-matched-DOM directly to pixels at the same position in video frame. In order to realize accurate positioning of UAV’s patrolling video sequence images, a precise polynomial-rectifying algorithm is further constructed.

The basic idea of the proposed precise polynomial-rectifying algorithm is to find out homologous regions in block-matched-DOM for regions in video frame, so as to figure out accurate rectification parameters for mapping video frame to block-matched-DOM. And finally, accurate positioning of video frame can be realized by using accurate rectification parameters to calculate geodetic coordinates of each pixel in video frame. It should be noted that, we are committed to find out homologous regions between video frame and block-matched-DOM, instead of finding out the homologous points. Because homologous regions are more stable and reliable than homologous points under numerous negative influences. Where, homologous regions refer to the most similar local areas between two images.

Through in-depth study of common characteristics between block-matched-DOM and video frame, the precise polynomial-rectifying algorithm is constructed based on three assumptions: (1) video frame and block-matched-DOM can be regarded as two adjacent sequence images. (2) Overall surface features are similar between video frame and block-matched-DOM. (3) Pixels in a local area of the video frame share a same deformation law.

#### Constructing polynomials of video frame and that of block-matched-DOM

In order to reduce negative influence of illumination variation, gradient magnitude images of video frame and that of block-matched-DOM are used for image matching. In order to further reduce negative influence of diverse surface specific features, gradient magnitude images of video frame and that of block-matched-DOM are represented by second-order polynomials respectively, and the second-order polynomials of these two images are used for image matching ultimately.

**Figure 4:**
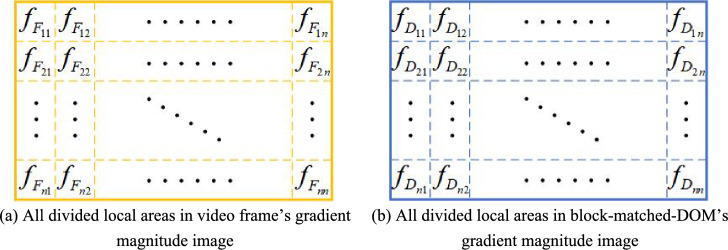
Local areas divided in video frame and block-matched-DOM.

As shown in Fig. 4, gradient magnitude images of video frame and that of block-matched-DOM are evenly divided into $n\times n$ local areas respectively, and each of the local areas is represented by a second-order polynomial as:

6 $$\left\{\begin{array}{l}{f}_{{F}_{ij}}({X}_{\rm I},{T}_{\rm I})={X}_{\rm I}^{T}{A}_{\rm I}{X}_{\rm I}+{{B}_{\rm I}}^{T}{X}_{\rm I}+{C}_{\rm I} \\ {f}_{{D}_{ij}}({X}_{\Pi },{T}_{\Pi })={X}_{\Pi }^{T}{A}_{\Pi }{X}_{\Pi }+{{B}_{\Pi }}^{T}{X}_{\Pi }+{C}_{\Pi }\end{array}\right.$$

where, ${f}_{{F}_{ij}}({X}_{\rm I},{T}_{\rm I})$ and ${f}_{{D}_{ij}}({X}_{\Pi },{T}_{\Pi })$ are intensity of local area of row $i$ and column $j$ in Fig. 4a,b respectively, $i=(1,\cdots ,n)$, $j=(1,\cdots ,n)$; ${X}_{\rm I}={({x}_{\rm I},{y}_{\rm I})}^{T}$, ${X}_{\Pi }={({x}_{\Pi },{y}_{\Pi })}^{T}$, $T$ represent transpose of a matrix (vector),$({x}_{\rm I},{y}_{\rm I})$ and $({x}_{\Pi },{y}_{\Pi })$ are pixel coordinates in local areas of Fig. 4a,b respectively; ${T}_{\rm I}$ and ${T}_{\Pi }$ are production time of video frame and that of block-matched-DOM respectively; $${A}_{\rm I}=\left(\begin{array}{cc}{m}_{4}^{\rm I}& {m}_{6}^{\rm I}/2\\ {m}_{6}^{\rm I}/2& {m}_{5}^{\rm I}\end{array}\right)$$, $${A}_{\Pi }=\left(\begin{array}{cc}{m}_{4}^{\Pi }& {m}_{6}^{\Pi }/2\\ {m}_{6}^{\Pi }/2& {m}_{5}^{\Pi }\end{array}\right)$$, ${A}_{\rm I}$ and ${A}_{\Pi }$ are second-order coefficient matrix of their polynomials respectively; ${B}_{\rm I}={({m}_{2}^{\rm I},{m}_{3}^{\rm I})}^{T}$,${B}_{\Pi }={({m}_{2}^{\Pi },{m}_{3}^{\Pi })}^{T}$, ${B}_{\rm I}$ and ${B}_{\Pi }$ are first-order coefficient vectors of their polynomials respectively; ${C}_{\rm I}={m}_{1}^{\rm I}$, ${C}_{\Pi }={m}_{1}^{\Pi }$, ${C}_{\rm I}$ and ${C}_{\Pi }$ are scalars of their polynomials respectively; ${m}_{1}^{\rm I}$, ${m}_{2}^{\rm I}$, ${m}_{3}^{\rm I}$, ${m}_{4}^{\rm I}$, ${m}_{5}^{\rm I}$, ${m}_{6}^{\rm I}$, ${m}_{1}^{\Pi }$, ${m}_{2}^{\Pi }$, ${m}_{3}^{\Pi }$, ${m}_{4}^{\Pi }$, ${m}_{5}^{\Pi }$, ${m}_{6}^{\Pi }$ are parameters of polynomials.

#### Constructing differential-difference polynomials

Block-matched-DOM is the best matching area for video frame on datum-DOM. However, there are still irregular motion displacements between homologous regions of these two images due to numerous negative factors. Therefore, finding out homologous regions of these two images is important for precise positioning of video frame.

Based on the assumption that video frame and block-matched-DOM can be regarded as two adjacent sequence images, the second-order polynomials of video frame and that of block-matched-DOM can also be regarded as two adjacent sequence images. And then, differential-difference polynomials can be constructed based on Eq. (6), and further can be rewritten by using Taylor expansion for $\Delta X$ to the first order derivative as:

7 $$\begin{aligned} d &= f_{D} \left( {X_{\Pi } ,T_{\Pi } } \right) - f_{F} \left( {X_{\rm I} ,T_{\rm I} } \right) \nonumber \\ &= f_{D} \left( {X_{\rm I} + \Delta X,T_{\Pi } } \right) - f_{F} \left( {X_{\rm I} ,T_{\rm I} } \right) \nonumber  \\ & = f_{D} \left( {X_{\rm I} ,T_{\Pi } } \right) + \frac{{\partial f_{D} \left( {X_{\rm I} ,T_{\Pi } } \right)}}{{\partial X_{\rm I} }}\Delta X - f_{F} \left( {X_{\rm I} ,T_{\rm I} } \right) \nonumber  \\ &= X_{\rm I}^{T} A_{\Pi } X_{\rm I} + B_{\Pi }^{T} X_{\rm I} + C_{\Pi } + 2X_{\rm I}^{T} A_{\Pi } \Delta X + B_{\Pi }^{T} \Delta X - (X_{\rm I}^{T} A_{\rm I} X_{\rm I} + B_{\rm I}^{T} X_{\rm I} + C_{\rm I} \nonumber  \\ &= X_{\rm I}^{T} \left( {A_{\Pi } - A_{\rm I} } \right)X_{\rm I} + \left( {B_{\Pi } - B_{\rm I} + 2A_{\Pi } \Delta X} \right)^{T} X_{\rm I} + \left( {C_{\Pi } + B_{\Pi }^{T} \Delta X - C_{\rm I} } \right)  \end{aligned}$$

where, ${f}_{F}({X}_{\rm I},{T}_{\rm I})$ and ${f}_{D}({X}_{\Pi },{T}_{\Pi })$ are intensity of the corresponding local areas in Fig. 4a,b respectively; ${X}_{\rm I}$ and ${X}_{\Pi }$ are pixel coordinates in local areas of Fig. 4a,b respectively; ${T}_{\rm I}$ and ${T}_{\Pi }$ are production time of video frame and that of block-matched-DOM respectively;$${A}_{\rm I}=\left(\begin{array}{cc}{m}_{4}^{\rm I}& {m}_{6}^{\rm I}/2\\ {m}_{6}^{\rm I}/2& {m}_{5}^{\rm I}\end{array}\right)$$, $${A}_{\Pi }=\left(\begin{array}{cc}{m}_{4}^{\Pi }& {m}_{6}^{\Pi }/2\\ {m}_{6}^{\Pi }/2& {m}_{5}^{\Pi }\end{array}\right)$$; ${B}_{\rm I}={({m}_{2}^{\rm I},{m}_{3}^{\rm I})}^{T}$,${B}_{\Pi }={({m}_{2}^{\Pi },{m}_{3}^{\Pi })}^{T}$; ${C}_{\rm I}={m}_{1}^{\rm I}$, ${C}_{\Pi }={m}_{1}^{\Pi }$; ${m}_{1}^{\rm I}$, ${m}_{2}^{\rm I}$, ${m}_{3}^{\rm I}$, ${m}_{4}^{\rm I}$, ${m}_{5}^{\rm I}$, ${m}_{6}^{\rm I}$, ${m}_{1}^{\Pi }$, ${m}_{2}^{\Pi }$, ${m}_{3}^{\Pi }$, ${m}_{4}^{\Pi }$, ${m}_{5}^{\Pi }$, ${m}_{6}^{\Pi }$ are parameters of polynomials; ${X}_{\Pi }={X}_{\rm I}+\Delta X$.

**Figure 5:**
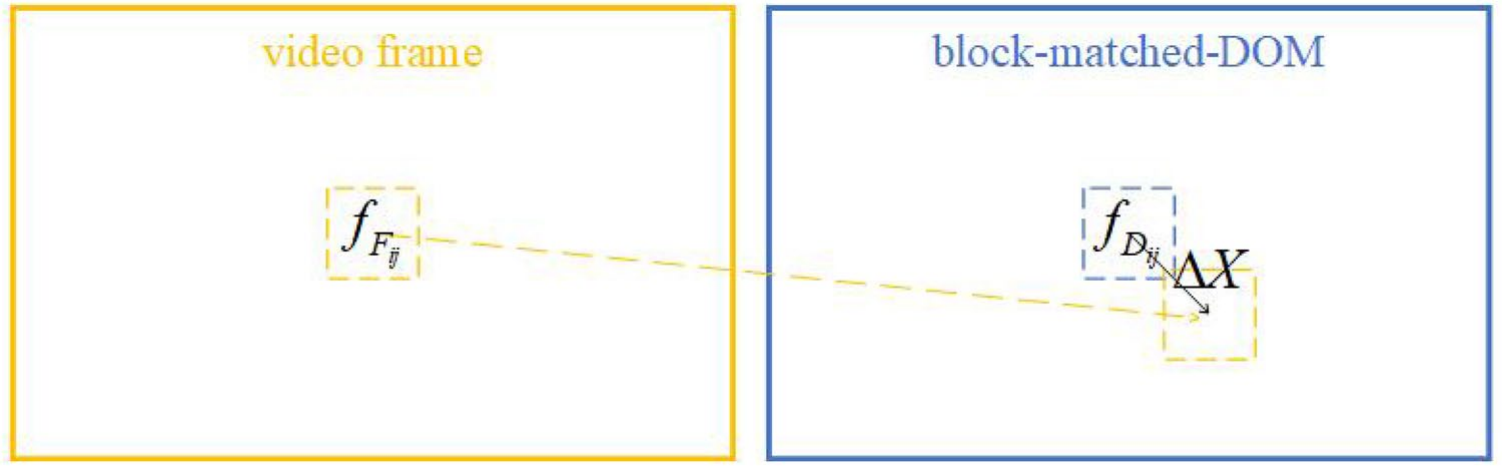
Small motion displacement from a local area of video frame to the corresponding local area of block-matched-DOM.

As shown in Fig. 5, $\Delta X$ is a small motion displacement from a local area of video frame to the corresponding local area of block-matched-DOM. That is to say, homologous regions between video frame and block-matched-DOM can be obtained by finding out $\Delta X$ that can minimizes $d$ in Eq. (7).

In Eq. (7), let $d$ be exactly equal to zero, we can obtain as:

8 $$\left\{\begin{array}{l}{A}_{\Pi }={A}_{\rm I} \\ {A}_{\Pi }\Delta X=({B}_{\rm I}-{B}_{\Pi })/2\\ {{B}_{\Pi }}^{T}\Delta X={C}_{\rm I}-{C}_{\Pi }\end{array}\right.$$

Further, we can obtain equations of $\Delta X$ as:

9 $${A}_{\Delta X}\Delta X={L}_{\Delta X}$$

where, $${A}_{\Delta X}=\left[\begin{array}{c}\left({A}_{\rm I}+{A}_{\Pi }\right)/2\\ {{B}_{\Pi }}^{T}\end{array}\right]$$; $${L}_{\Delta X}=\left[\begin{array}{c}({B}_{\rm I}-{B}_{\Pi })/2\\ {C}_{\rm I}-{C}_{\Pi }\end{array}\right]$$.

#### Constructing precise rectifying equations

$\Delta X$ In Eq. (9) can be also regarded as registration errors between video frame and block-matched-DOM. These registration errors are supposed to be caused by video frame’s scaling, displacement, rotation, distortion and etc. And then, $\Delta X$ can be also represented by second-order polynomials as:

10 $$\Delta X=\left(\begin{array}{c}{a}_{0}+{a}_{1}{x}_{f}+{a}_{2}{y}_{f}+{a}_{3}{x}_{f}^{2}+{a}_{4}{x}_{f}{y}_{f}+{a}_{5}{y}_{f}^{2}\\ {b}_{0}+{b}_{1}{x}_{f}+{b}_{2}{y}_{f}+{b}_{3}{x}_{f}^{2}+{b}_{4}{x}_{f}{y}_{f}+{b}_{5}{y}_{f}^{2}\end{array}\right)$$

where, $\left({x}_{f},{y}_{f}\right)$ are coordinates of a local area in video frame; ${a}_{0}$, ${a}_{1}$, ${a}_{2}$, ${a}_{3}$, ${a}_{4}$, ${a}_{5}$, ${b}_{0}$, ${b}_{1}$, ${b}_{2}$, ${b}_{3}$, ${b}_{4}$, ${b}_{5}$ are parameters of polynomials.

According to Eqs. (9) and (10), precise rectifying equations can be constructed ultimately as:

11 $$At=L$$

Where, $$A={A}_{\Delta X}\left(\begin{array}{c}1,{x}_{f},{y}_{f},{x}_{f}^{2},{x}_{f}{y}_{f},{y}_{f}^{2},\mathrm{0,0},\mathrm{0,0},\mathrm{0,0}\\ \mathrm{0,0},\mathrm{0,0},\mathrm{0,0},1,{x}_{f},{y}_{f},{x}_{f}^{2},{x}_{f}{y}_{f},{y}_{f}^{2}\end{array}\right)$$, $${A}_{\Delta X}=\left[\begin{array}{c}\left({A}_{\rm I}+{A}_{\Pi }\right)/2\\ {{B}_{\Pi }}^{T}\end{array}\right]$$, ${x}_{f}$ and ${y}_{f}$ are column and row numbers of a local area in video frame respectively;$t={({a}_{0},{a}_{1},{a}_{2},{a}_{3},{a}_{4},{a}_{5},{b}_{0},{b}_{1},{b}_{2},{b}_{3},{b}_{4},{b}_{5})}^{T}$, $t$ is a vector of unknown parameters to be resolved; $$L=\left[\begin{array}{c}({B}_{\rm I}-{B}_{\Pi })/2\\ {C}_{\rm I}-{C}_{\Pi }\end{array}\right]$$.

#### Constructing optimal estimation model

As shown in Eq. (11), the task of finding out $\Delta X$ is converted to find out $t$, and each pair of local areas in Fig. 4a,b can construct 3 equations. That is to say, $3{n}^{2}$ equations can be constructed in the form of Eq. (11), as there are ${n}^{2}$ pairs of local areas in Fig. 4a,b.

According to the presumption that the minimum energy difference should exist between video frame and block-matched-DOM in homologous regions, the optimization criteria for the $3{n}^{2}$ equations that are constructed in the form of Eq. (11) can be proposed as:

12 $$\underset{arg \,\,\,\,t}{min}{V}^{T}\Omega V$$

where, $V=At-L$, $V$ is a vector of residual errors; $\Omega$ is a weight matrix;$t$ is a vector of unknown parameters; Meaning of the remaining parameters refer to Eq. (11).

In order to obtain the optimal estimation of $t$, following iteration process are recommended.

① Down-sample images and construct k-layer image pyramids for video frame and block-matched-DOM.

② Set $\Omega =I$, $I$ is an identity matrix; Set $i=k$ and $t={\left(\mathrm{0,0},\mathrm{0,0},\mathrm{0,0},\mathrm{0,0},\mathrm{0,0},\mathrm{0,0}\right)}^{T}$.

③ Construct matrix $A$ and $L$ according to the ith layer images of pyramid.

④ Calculate correction vector for $t$ as: $\Delta t={({A}^{T}\Omega A)}^{-1}{A}^{T}\Omega (L-A{t}_{0})$.

⑤ Calculate vector of residual errors as: $V=A\left({t}_{0}+\Delta t\right)-L$.

⑥ Redefine weight matrix as: $$\Omega =\left(\begin{array}{ccc}{\Omega }_{1}& 0& \begin{array}{cc}\dots & 0\end{array}\\ 0& {\Omega }_{2}& \begin{array}{cc}\cdots & 0\end{array}\\ \begin{array}{c}\vdots \\ 0\end{array}& \begin{array}{c}\vdots \\ 0\end{array}& \begin{array}{c}\begin{array}{cc}\ddots & \vdots \end{array}\\ \begin{array}{cc}0& {\Omega }_{n}\end{array}\end{array}\end{array}\right)$$, $${\Omega }_{j}=\left\{\begin{array}{c}{\Omega }_{j}, \left|{V}_{j}\right|\le 1.5\sigma \\ {\Omega }_{j}\frac{{\sigma }_{e}}{\left|{e}_{j}\right|}, 1.5\sigma <\left|{V}_{j}\right|\\ 0, 3\sigma <\left|{V}_{j}\right|\end{array}\right.\le 3\sigma$$, $\sigma =\sqrt{\frac{{V}^{T}\Omega V}{{n}^{2}-12}}$, $j=1,\cdots ,n$.

⑦ Set $$t=t+\Delta t$$.

⑧ Repeat steps ④–⑦ $m$ times, and we set $m=3$ in this study.

⑨ Set $i=k-1$. Repeat steps ③–⑧ until $i$ equals zero, and the optimal estimates of $t$ is calculated out from the last iteration.

### Positioning of UAV’s patrolling video frame

By using the optimal estimates of $t$ above resolved, precise geodetic coordinates of each pixel in video frame can be obtained as below:

13 $$\left(\begin{array}{c}L\\ B\end{array}\right)=P\widehat{X}$$

where, $(L,B)$ are geodetic coordinates of a pixel in video frame;$$P=\left(\begin{array}{ccc}{P}_{A}& {P}_{B}& {P}_{C}\\ {P}_{D}& {P}_{E}& {P}_{F}\end{array}\right)$$, $P$ is a transformation matrix provided by producer of region-DOM; $\widehat{X}=X+{X}_{f}t$; $X={\left(x,y,1\right)}^{T}$, $\left(x,y\right)$ are pixel coordinates of a pixel in video frame; $${X}_{f}=\left(\begin{array}{c}1,{x}_{f},{y}_{f},{x}_{f}^{2},{x}_{f}{y}_{f},{y}_{f}^{2},\mathrm{0,0},\mathrm{0,0},\mathrm{0,0}\\ \begin{array}{c}\mathrm{0,0},\mathrm{0,0},\mathrm{0,0},1,{x}_{f},{y}_{f},{x}_{f}^{2},{x}_{f}{y}_{f},{y}_{f}^{2}\\ \mathrm{0,0},\mathrm{0,0},\mathrm{0,0},\mathrm{0,0},\mathrm{0,0},\mathrm{0,0}\end{array}\end{array}\right)$$, ${x}_{f}$ and ${y}_{f}$ are column and row numbers of the local area where the pixel is located; $t$ is the optimal rectifying parameters calculated above.

Finally, according to Eq. (13), precise positioning of UAV’s patrolling video sequence images can be realized by calculating geodetic coordinates of each pixel in UAV’s patrolling video sequence images.

## Case study

Three practical experiments are designed in this study, which includes 3 videos and 2 region-DOMs. Among them, 3 videos are shot by 3 sorties fly of UAV in different areas, including town area, river area and high relief amplitude area. 2 region-DOMs have different spatial resolutions, one of the 2 region-DOMs has a lower spatial resolution, and the other one has a higher spatial resolution.

### The first experiment

**Figure 6:**
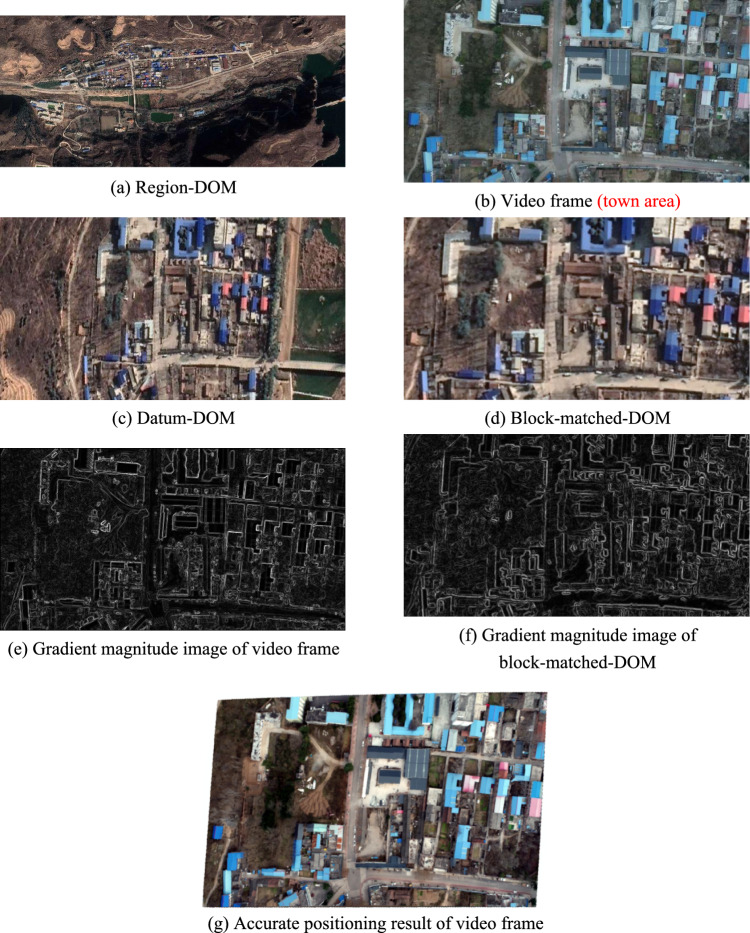

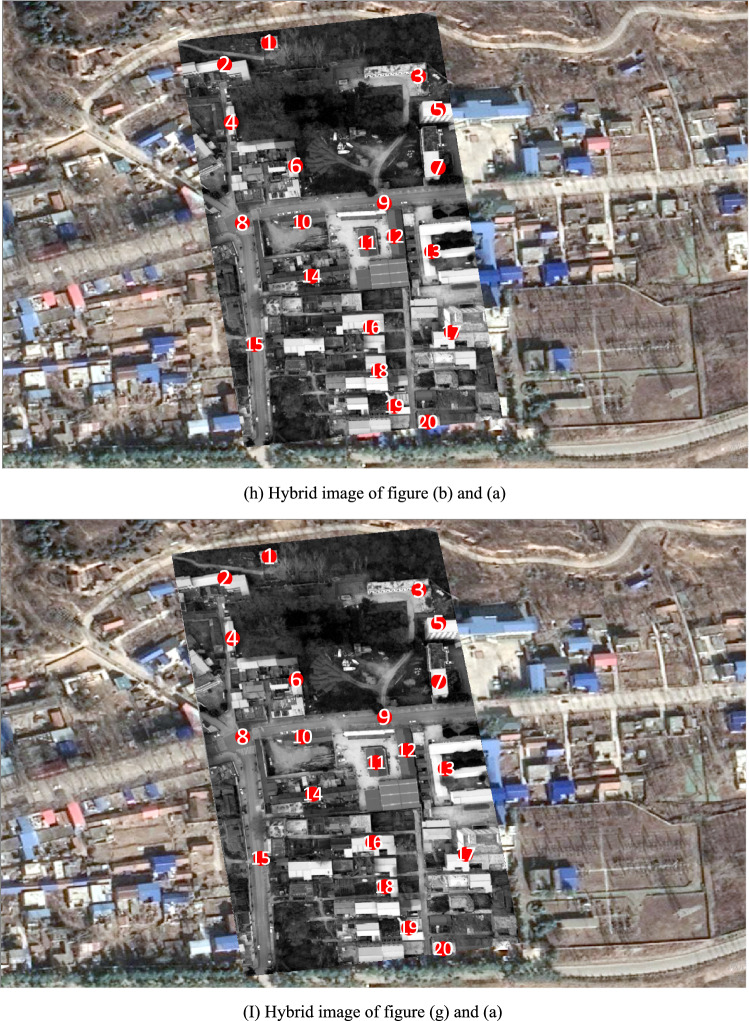
The first experiment.

As shown in Fig. 6, Fig. 6a is region-DOM used in this experiment, which was made on January 31, 2020, with length of 4096 pixels, width of 1792 pixels, and spatial resolution of 0.493663 m/pixels. Video used in this experiment was shot by 1 sortie fly of UAV at an altitude of about 250 m on November 24, 2021, including 3154 frames, with fps (frames per second) of 23.98, spatial resolution of 0.0684932 m/pixels, length of 4096 pixels and width of 2160 pixels in a single frame. In addition, the video was shot in town area. Figure 6b is the 301st frame of the video used in this experiment, and is picked out for algorithm demonstration without loss of generality. POS data of the 301st frame are obtained by IMU (Inertial Measurement Unit) mounted on UAV, where, the center geodetic coordinates are (111.2661504°, 34.2428275°), flight altitude is 250.1 m, pitch angle is − 8.3°, roll angle is − 1.3°, and yaw angle is 82.5°.

According to the theory proposed in section "Extraction of datum-DOM", Fig. 6c is datum-DOM that is extracted from Fig. 6a on the basis of POS data of Fig. 6b.

According to the theory proposed in section "Construction of robust block-matching algorithm", Fig. 6d is block-matched-DOM that is extracted from Fig. 6c, and Fig. 6d is the best matching area for Fig. 6b on Fig. 6c. And by timekeeping in the program,

According to the theory proposed in section "construction of precise polynomial-rectifying algorithm", Fig. 6e,f are gradient magnitude images that are calculated from Fig. 6b,d respectively. And, the optimal estimation $t$ calculated out from Fig. 6e,f is, $t=\left(-7.8973,0.1650,-0.0083,0.0002,0.0004,-0.0002,2.1185,-0.1622,0.2590,0.0003,0.0006,0.0017\right)$.

According to the theory proposed in section "Positioning of UAV’s patrolling video frame", Fig. 6g is the accurate positioning result of video frame. Figure 6g is obtained by using parameter $t$ and $P$ to calculate geodetic coordinates of each pixel in Fig. 6b. Where, $t$ is obtained by optimal estimation model mentioned above, $P$ is provided by producer of region-DOM, and $$P=\left(\begin{array}{ccc}0.0000053644& 0& 111.2558010221\\ 0& -0.0000053644& 34.2457553744\end{array}\right)$$.

Figure 6h is a hybrid image formed by superimposing Fig. 6b on Fig. 6a according to their geodetic coordinates. Where, geodetic coordinates of Fig. 6a are pre-acquired, and geodetic coordinates of Fig. 6b are directly assigned from the block-matched-DOM. Among Fig. 6h, the gray area is Fig. 6b and the 20 red points are interest points on Fig. 6b. Distance deviations between the 20 red homologous points in Fig. 6a,b are measured in ArcGIS and listed in Table 1, and the average distance deviation is 4.614 m.

**Table 1:** Distance deviations between 20 red homologous points in Fig. 6h,i.

Point number	1	2	3	4	5	6	7	8	9	10
Deviation of homologous points in Fig. 6h/m	2.834	1.807	1.622	1.942	5.698	3.149	5.511	3.537	4.133	3.111
Deviation of homologous points in Fig. 6i/m	1.234	3.307	1.268	2.519	5.517	2.735	2.523	1.184	0.555	1.857
Point number	11	12	13	14	15	16	17	18	19	20
Deviation of homologous points in Fig. 6h/m	5.729	5.082	5.537	3.617	4.802	5.956	8.365	6.487	6.587	6.764
Deviation of homologous points in Fig. 6i/m	1.638	1.491	1.128	1.303	0.746	1.409	6.489	2.154	2.096	2.287
Mean deviation of homologous points in Fig. 6h/m	4.614									
Mean deviation of homologous points in Fig. 6i/m	2.172									

Figure 6i is a hybrid image formed by superimposing Fig. 6g on Fig. 6a according to their geodetic coordinates. Where, geodetic coordinates of Fig. 6a are pre-acquired, and geodetic coordinates of Fig. 6g are obtained by using parameter $t$ and $P$ to calculate geodetic coordinates of each pixel in video frame. Among Fig. 6i, the gray area is Fig. 6g and the 20 red points are interest points on Fig. 6b. In order to improve reliability and generality of the experiment, all the 20 red homologous points are evenly selected from distinctive terrain points and building points without any deliberate adjustment. Distance deviations between the 20 red homologous points in Fig. 6a,g are measured in ArcGIS and listed in Table 1, and the average distance deviation is 2.172 m.

By timekeeping in our program, it takes about 0.206 s to complete extracting of the block-matched-DOM, it takes about 0.330 s to complete calculating of the optimal estimation $t$, and it takes about 0.101 s to complete calculating of the precise geodetic coordinates of video frame pixel by pixel. That is to say, the total positioning time of this UAV’s patrolling video frame is less than 1 s.

### The second experiment

**Figure 7:**
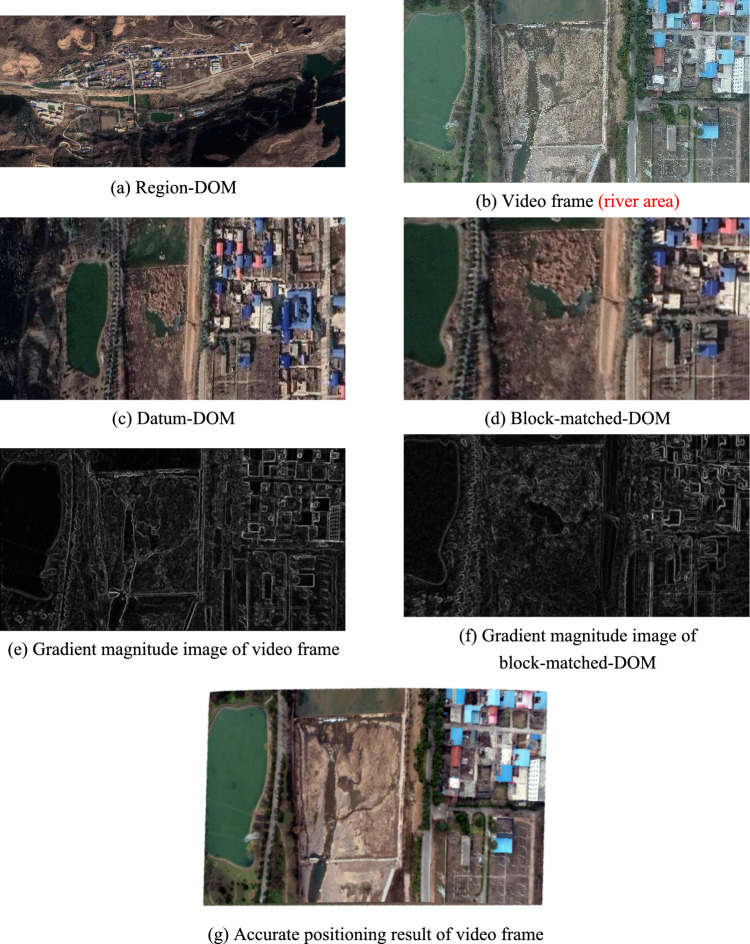

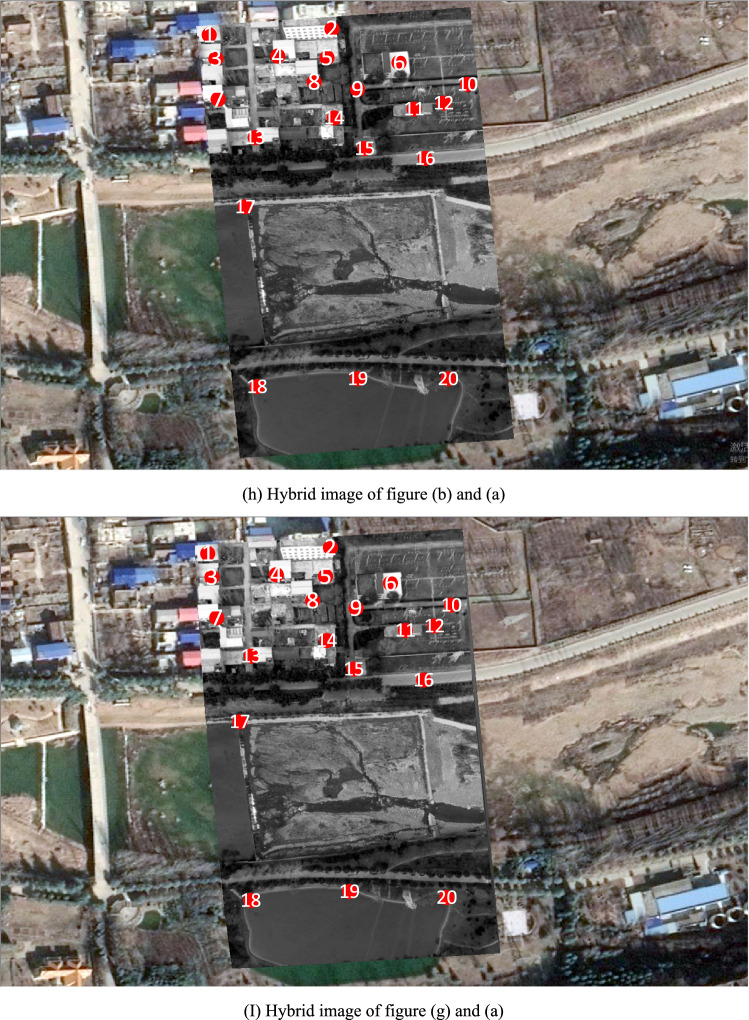
The second experiment.

As shown in Fig. 7, Fig. 7a is region-DOM used in this experiment, and is same as Fig. 6a. Video used in this experiment was shot by 1 sortie fly of UAV at an altitude of about 250 m on November 25, 2021, including 4687 frames, with fps (frames per second) of 23.98, spatial resolution of 0.0684932 m/pixels, length of 4096 pixels and width of 2160 pixels in a single frame. In addition, the video was shot in river area. Figure 7b is the 3547st frame of the video used in this experiment, and is picked out for algorithm demonstration without loss of generality. POS data of the 3547st frame are obtained by IMU mounted on UAV, where, the center geodetic coordinates are (111.2658703°, 34.2406338°), flight altitude is 250.1 m, pitch angle is − 7.1°, roll angle is 2.9°, and yaw angle is − 94.8°.

According to the theory proposed in section "Extraction of datum-DOM", Fig. 7c is datum-DOM that is extracted from Fig. 7a on the basis of POS data of Fig. 7b.

According to the theory proposed in section "construction of robust block-matching algorithm", Fig. 7d is block-matched-DOM that is extracted from Fig. 7c,d is the best matching area for Fig. 7b on Fig. 7c.

According to the theory proposed in section "Construction of precise polynomial-rectifying algorithm", Fig. 7e,f are gradient magnitude images that are calculated from Fig. 7b,d respectively. And, the optimal estimation $\mathrm{t}$ calculated out from Fig. 7e,f is, $t=\left(-17.6265,0.0919,-0.0814,0.0004,0.0001,0.0001,-36.3304,0.0845,0.0535,0.0005,0.0000,-0.0006\right)$.

According to the theory proposed in Section "Positioning of UAV’s patrolling video frame", Fig. 7g is the accurate positioning result of video frame. Figure 7g is obtained by using parameter $t$ and $P$ to calculate geodetic coordinates of each pixel in Fig. 7b. Where, $t$ is obtained by optimal estimation model mentioned above, $P$ is provided by producer of region-DOM, and $$P=\left(\begin{array}{ccc}0.0000053644& 0& 111.2558010221\\ 0& -0.0000053644& 34.2457553744\end{array}\right)$$.

Figure 7h is a hybrid image formed by superimposing Fig. 7b on Fig. 7a in software according to their geodetic coordinates. Where, geodetic coordinates of Fig. 7a are pre-acquired, and geodetic coordinates of Fig. 7b are directly assigned from the block-matched-DOM. Among Fig. 7h, the gray area is Fig. 7b and the 20 red points are interest points on Fig. 7b. Distance deviations between the 20 red homologous points in Fig. 7a,b are measured in ArcGIS and listed in Table 2, and the average distance deviation is 5.240 m.

**Table 2:** Distance deviations between 20 red homologous points in Fig. 7h,i.

Point number	1	2	3	4	5	6	7	8	9	10
Deviation of homologous points in Fig. 7h/m	6.262	5.44	6.344	5.725	7.465	3.538	5.698	5.383	6.675	5.501
Deviation of homologous points in Fig. 7i/m	1.602	7.240	1.556	1.118	1.622	1.831	3.778	1.478	1.646	1.029
Point number	11	12	13	14	15	16	17	18	19	20
Deviation of homologous points in Fig. 7h/m	3.728	4.763	5.720	5.305	4.763	3.406	4.622	6.152	4.051	4.262
Deviation of homologous points in Fig. 7i/m	0.539	0.354	2.157	0.720	0.411	0.307	0.219	5.540	5.811	6.106
Mean deviation of homologous points in Fig. 7h/m	5.240									
Mean deviation of homologous points in Fig. 7i/m	2.253									

Figure 7i is a hybrid image formed by superimposing Fig. 7g on Fig. 7a according to their geodetic coordinates. Where, geodetic coordinates of Fig. 7a are pre-acquired, and geodetic coordinates of Fig. 7g are obtained by using parameter $t$ and $P$ to calculate geodetic coordinates of each pixel in video frame. Among Fig. 7i, the gray area is Fig. 7g and the 20 red points are interest points on Fig. 7b. In order to improve reliability and generality of the experiment, all the 20 red homologous points are evenly selected from distinctive terrain points and building points without any deliberate adjustment. Distance deviations between the 20 red homologous points in Fig. 7a,g are measured in ArcGIS and listed in Table 2, and the average distance deviation is 2.253 m.

By timekeeping in our program, it takes about 0.119 s to complete extracting of the block-matched-DOM, it takes about 0.118 s to complete calculating of the optimal estimation $t$, and it takes about 0.053 s to complete calculating of the precise geodetic coordinates of video frame pixel by pixel. That is to say, the total positioning time of this UAV’s patrolling video frame is less than 1 s.

### The third experiment

**Figure 8:**
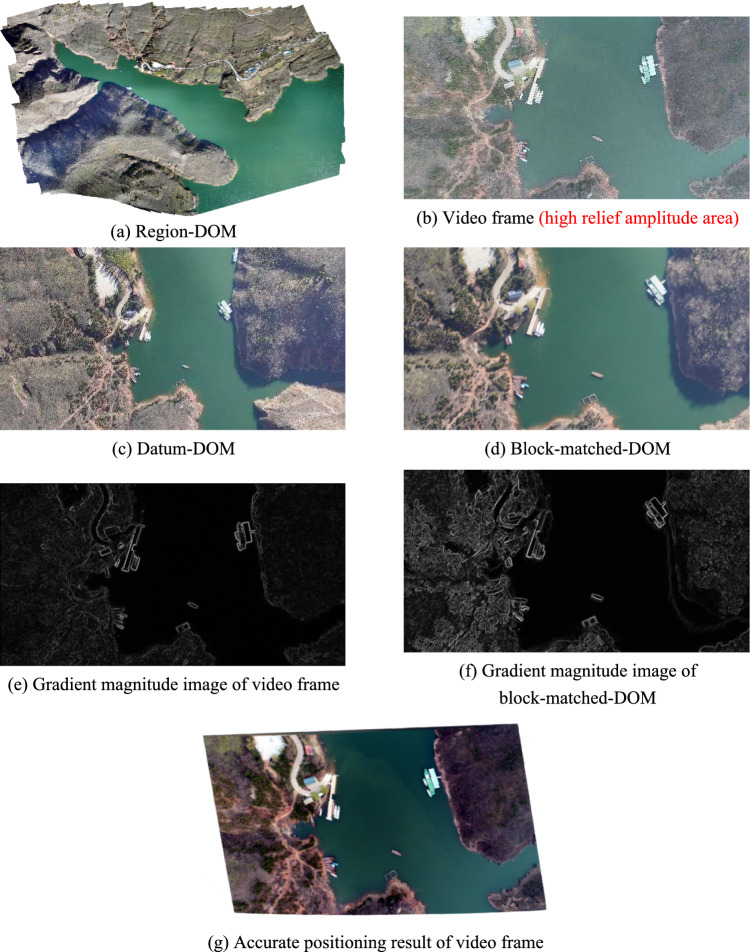

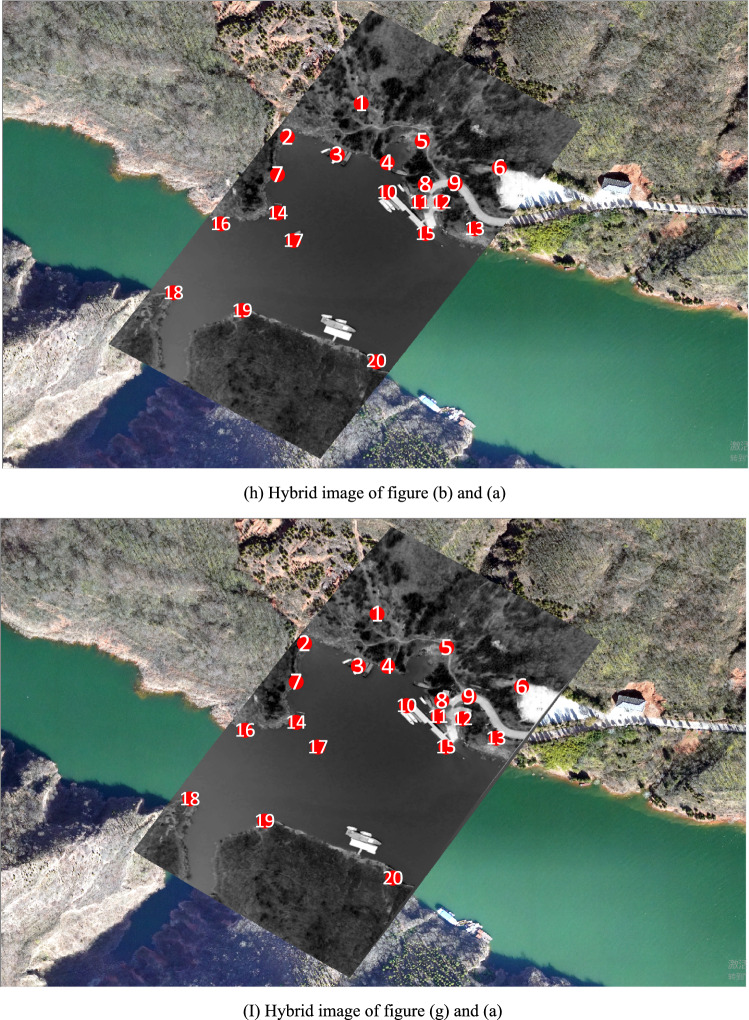
The third experiment.

As shown in Fig. 8, Fig. 8a is region-DOM used in this experiment, which was made on May 13, 2021, with length of 19,266 pixels, width of 14,483 pixels, and spatial resolution of 0.08 m/pixels. Video used in this experiment was shot by 1 sortie fly of UAV at an altitude of about 250 m on November 26, 2021, including 3788 frames, with fps (frames per second) of 23.98, spatial resolution of 0.0684932 m/pixels, length of 4096 pixels and width of 2160 pixels in a single frame. In addition, the video was shot in high relief amplitude area. Figure 8b is the 901st frame of the video used in this experiment, and is picked out for algorithm demonstration without loss of generality. POS data of the 901st frame are obtained by IMU mounted on UAV, where, the center geodetic coordinates are (111.2504477°, 34.2280547°), flight altitude is 250.5 m, pitch angle is − 22.7°, roll angle is − 9.7°, and yaw angle is 123.2°.

According to the theory proposed in section "Extraction of datum-DOM", Fig. 8c is datum-DOM that is extracted from Fig. 8a on the basis of POS data of Fig. 8b.

According to the theory proposed in section "construction of robust block-matching algorithm", Fig. 8d is block-matched-DOM that is extracted from Fig. 8c,d is the best matching area for Fig. 8b on Fig. 8c.

According to the theory proposed in section "Construction of precise polynomial-rectifying algorithm", Fig. 8e,f are gradient magnitude images that are calculated from Fig. 8b,d respectively. And, the optimal estimation $\mathrm{t}$ calculated out from Fig. 8e,f is, $\mathrm{t}=\left(-1.3754,0.0206,-0.1524,0.0006,-0.0004,-0.0001,6.9715,-0.0663,0.2922,-0.0006,0.0008,0.0009\right)$.

According to the theory proposed in Section "Positioning of UAV’s patrolling video frame", Fig. 8g is the accurate positioning result of video frame. Figure 8g is obtained by using parameter $\mathrm{t}$ and $\mathrm{P}$ to calculate geodetic coordinates of each pixel in Fig. 8b. Where, $\mathrm{t}$ is obtained by optimal estimation model mentioned above, $\mathrm{P}$ is provided by producer of region-DOM, and $$\mathrm{P}=\left(\begin{array}{ccc}0.0000008683& 0& 111.2441313977\\ 0& -0.0000007212& 34.2315524404\end{array}\right)$$.

Figure 8h is a hybrid image formed by superimposing Fig. 8b on Fig. 8a according to their geodetic coordinates. Where, geodetic coordinates of Fig. 8a are pre-acquired, and geodetic coordinates of Fig. 8b are directly assigned from the block-matched-DOM. Among Fig. 8h, the gray area is Fig. 8b and the 20 red points are interest points on Fig. 8b. Distance deviations between the 20 red homologous points in Fig. 8a,b are measured in ArcGIS and listed in Table 3, and the average distance deviation is 7.105 m.

**Table 3:** Distance deviations between 20 red homologous points in Fig. 8h,i.

Point number	1	2	3	4	5	6	7	8	9	10
Deviation of homologous points in Fig. 8h/m	8.594	12.119	8.255	8.470	8.483	2.828	9.881	5.656	6.587	5.497
Deviation of homologous points in Fig. 8i/m	1.431	4.612	2.136	3.272	4.114	0.358	3.967	0.870	1.485	1.69
Point number	11	12	13	14	15	16	17	18	19	20
Deviation of homologous points in Fig. 8h/m	4.460	5.352	3.039	7.484	4.483	10.303	7.137	10.812	6.426	6.236
Deviation of homologous points in Fig. 8i/m	0.400	0.738	1.204	4.968	0.684	6.99	3.66	11.339	9.921	8.547
Mean deviation of homologous points in Fig. 8h/m	7.105									
Mean deviation of homologous points in Fig. 8i/m	3.619									

Figure 8i is a hybrid image formed by superimposing Fig. 8g on Fig. 8a according to their geodetic coordinates. Where, geodetic coordinates of Fig. 8a are pre-acquired, and geodetic coordinates of Fig. 8g are obtained by using parameter $\mathrm{t}$ and $\mathrm{P}$ to calculate geodetic coordinates of each pixel in video frame. Among Fig. 8i, the gray area is Fig. 8g and the 20 red points are interest points on Fig. 8b. In order to improve reliability and generality of the experiment, all the 20 red homologous points are evenly selected from distinctive terrain points and building points without any deliberate adjustment. Distance deviations between the 20 red homologous points in Fig. 8a,g are measured in ArcGIS and listed in Table 3, and the average distance deviation is 3.619 m.

By timekeeping in our program, it takes about 0.118 s to complete extracting of the block-matched-DOM, it takes about 0.122 s to complete calculating of the optimal estimation $t$, and it takes about 0.074 s to complete calculating of the precise geodetic coordinates of video frame pixel by pixel. That is to say, the total positioning time of this UAV’s patrolling video frame is less than 1 s.

### Experimental analysis

In the first experiment, spatial resolution of region-DOM is far less than that of video frame, region-DOM’s surface universal features are similar with video frame’s, and region-DOM’s surface specific features and illumination condition are great different from video frame’s. From the experimental results, we can see that average positioning deviation of all interest points in Fig. 6h is about 4.614 m, and average positioning deviation of all interest points in Fig. 6i is about 2.172 m. Among them, interest points that are located on roads and low-rise buildings have lower positioning deviations, while interest points that are located on high-rise buildings have higher positioning deviations.

In the second experiment, spatial resolution of region-DOM is still far less than that of video frame, region-DOM’s surface universal features are similar with video frame’s, region-DOM’s surface specific features and illumination condition are greatly different from video frame’s, and surface features on the left side of video frame is significantly less than those on the right side. From the experimental results, we can see that average positioning deviation of all interest points in Fig. 7h is about 5.2402 m, and average positioning deviation of all interest points in Fig. 7i is about 2.2532 m. Among them, interest points that are located on roads and low-rise buildings have lower positioning deviations, interest points that are located on high-rise buildings have higher positioning deviations, and interest points that are located on the left side of video frame have higher positioning deviations than those on the right side.

In the third experiment, spatial resolution of region-DOM is similar with that of video frame, region-DOM’s surface universal features are similar with video frame’s, region-DOM’s surface specific features and illumination condition are a little different from video frame’s, while there are extensive mountain body shadows on region-DOM. From the experimental results, we can see that average positioning deviation of all interest points in Fig. 8h is about 7.1051 m, and average positioning deviation of all interest points in Fig. 8i is about 3.6193 m. Among them, interest points that are located on roads and low-rise buildings have lower positioning deviations than the first two experiments, while interest points that are located on mountain edges have the highest positioning deviations.

By analyzing the above three experiments, following conclusions can be achieved.

1. Geometrical shape of video frame deformed obviously after accurate positioning, as shown in Figs. 6g, 7g and 8g.
2. The average positioning deviations of video frame by using the proposed robust bock-matching algorithm is 5.653 m, and the average positioning deviations of video frame by using the proposed precise polynomial-rectifying algorithm is 2.681 m. That is to say, positioning accuracy of video frame can be significantly increased by using the proposed precise polynomial-rectifying algorithm.
3. The red homologous points located on roads and low-rise buildings have a higher positioning accuracy, while the red homologous points located on mountains and high-rise buildings have a lower positioning accuracy.
4. Using region-DOM of high spatial resolution can significantly improve positioning accuracy of video frame, while extensive shadows that are similar to video frame’s surface universal features will significantly decrease positioning accuracy of video frame.
5. The proposed model can be applied in various areas, such as, town area, river area, high relief amplitude area and etc. And experiment results show that, the average positioning accuracy in town area and river area is gentle higher than that in high relief amplitude area, as high terrain relief will impose a negative influence on the distortion of imaging.
6. By timekeeping in our program, the average time of extracting the block-matched-DOM is about 0.148 s, the average time of calculating the optimal estimation $t$ is about 0.19 s, and the average time of calculating all pixels’ precise geodetic coordinates in a video frame is about 0.076 s. That is to say, the total positioning time of a UAV’s patrolling video frame is less than 1 s.
7. The proposed methods can be also applied in the field of medical image registration, remote sensing image registration, visual navigation of other industries and etc. Subsequently, the current mathematical model can be optimized significantly by fusing with multi-source data, such as airborne LiDAR point cloud, and then can achieve a higher positioning accuracy and a broader application.

## Conclusion

In order to realize real-time positioning of UAV’s patrolling video sequence images, a visual positioning model is recommended, including a robust block-matching algorithm and a precise polynomial-rectifying algorithm.

First, the robust block-matching algorithm is constructed to realize roughly positioning of UAV’s video patrolling video sequence images. The robust block-matching algorithm is divided into 5 steps, including scaling datum-DOM, block-matching roughly based on RGB, Block-matching roughly based on gradient magnitude, block-matching robustly, and extracting block-matched-DOM. Through the above 5 steps, the so-called block-matched-DOM can be obtained, and rough positioning of UAV’s patrolling video sequence images can be realized by assigning geodetic coordinates of each pixel in block-matched-DOM to pixels at the same position in UAV’s patrolling video sequence images.

Second, the precise polynomial-rectifying algorithm is constructed to realize accurate positioning of UAV’s patrolling video sequence images. The precise polynomial-rectifying algorithm is divided into 5 steps, including constructing polynomials of video frame and that of block-matched-DOM, constructing differential-difference polynomials, constructing precise rectifying equations, constructing optimal estimation model, and calculating geodetic coordinates of interest points in video frame. Through the above 5 steps, the so-called accurate rectification parameters can be obtained, and accurate positioning of UAV’s patrolling video sequence images can be realized by using accurate rectification parameters to calculate geodetic coordinates of each pixel in UAV’s patrolling video sequence images.

Finally, all the proposed algorithms are verified by three practical experiments, and results indicate that the proposed robust block-matching algorithm can realize positioning of UAV’s patrolling video sequence images with an average accuracy of 5 m, even if spatial resolution, surface specific features, illumination and topographic relief of region-DOM are greatly different from that of UAV’s patrolling video sequence images. The proposed precise polynomial-rectifying algorithm can further improve positioning accuracy of UAV’s patrolling video sequence images with an average accuracy of about 2.5 m. And calculation time of positioning a single UAV’s patrolling video sequence image is less than 1 s.

## Data Availability

The data presented in this study are available on request from the corresponding author. The data are not publicly available due to another study related to this data is not yet publicly available.
